# Aphasia and Chronic Subdural Hematoma Evacuation: A Retrospective Cohort Study

**DOI:** 10.1227/neuprac.0000000000000148

**Published:** 2025-07-10

**Authors:** Surya Patil, John J. Francis, Michelot Michel, Takuma Maeda, Anand Veeravagu, David Bonda, Peyton L. Nisson

**Affiliations:** ‡ Department of Neurosurgery, Cedars-Sinai, Los Angeles, California, USA;; § School of Medicine, Case Western Reserve University, Cleveland, Ohio, USA;; ‖ College of Medicine, University of Florida, Gainesville, Florida, USA;; ¶ Department of Translational Neuroscience, Barrow Neurological Institute, Phoenix, Arizona, USA;; # Department of Neurosurgery, Stanford, Palo Alto, California, USA

**Keywords:** Chronic subdural hematoma, Subdural hematoma evacuation, Aphasia, Recovery, Risk factors

## Abstract

**BACKGROUND AND OBJECTIVES:**

The incidence of subdural hematomas (SDH) is expected to climb precipitously in the coming decades. Aphasia is one of the most common operative neurological symptoms of left-sided SDH. However, the rates of aphasia recovery after SDH evacuation have not been reported as neurological outcomes have been limited to mostly functional assessment scores and mortality. This study represents the first detailed analysis on aphasia and recovery in patients undergoing chronic SDH (cSDH) evacuation.

**METHODS:**

Adult patients who underwent evacuation of subacute or cSDH at a tertiary academic medical center between November 2013 and December 2021 were retrospectively identified using ICD 9 and 10 billing codes. Patients were categorized by the presence or absence of aphasia at initial presentation. Other clinical and demographic variables were also collected. After surgical evacuation, improvement and resolution of aphasia was recorded at the time of discharge, along with several outcome metrics.

**RESULTS:**

Of the 311 patients requiring cSDH evacuation who met inclusion criteria, 10% presented with aphasia. Risk factors for the development of aphasia were evaluated, including age, sex, hypertension, SDH size, location laterality, and midline shift size. Only left-sided SDH laterality was associated with a significantly greater risk of aphasia compared with right-sided and bilateral SDH (odds ratio 4.89, *P* < .001) while adjusting for age and sex. No difference in the rate of postoperative complications, neurological outcome, or mortality was found between patient cohorts. After surgical evacuation, 90% of patients had improvement of aphasia by the time of discharge, and 73% had complete resolution. At most recent follow-up (<180 days), aphasia had resolved in 83% of patients.

**CONCLUSION:**

This study represents one of the first detailed investigations into patients presenting with aphasia in the setting of cSDH. These findings provide unique insights to aid in management and rehabilitation planning of patients with cSDH presenting with aphasia.

**ABBREVIATIONS:**
cSDH: Chronic subdural hematomas
GCS: Glasgow Coma Scale
SDH: Subdural hematoma
SLP: Speech-language pathologist
TNDs: Transient neurological deficits.

The incidence of chronic subdural hematomas (cSDH) has increased significantly in recent years.^1^ This is likely a multifactorial phenomenon attributed to an aging population, rise in anticoagulant use, improvement in diagnostic tools and screening, and a higher incidence of traumatic brain injury-related incidents.^2-5^ According to the U.S. Census Bureau, by 2030, 20% of the U.S. population will be older than 65 years.^6^ This demographic shift can similarly be observed globally, with the United Nations projecting that by 2050, 1.5 billion individuals worldwide will be older than 65, with those aged 80 and older representing the fastest growing segment in developed countries.^7^

Elderly patients are particularly at risk of subdural hematoma (SDH)-related morbidity and mortality, because of factors such as increased use of antithrombotic medications, rising rates of neurodegenerative diseases such as Alzheimer's, and cerebral atrophy.^8^ cSDH are typically located along the convexity surface of the cerebrum and can present with a wide range of symptoms. Some of these can include headache, seizures, imbalance, lethargy, limb weakness, numbness, and aphasia.^9,10^ Despite the hematoma often spanning across the cerebral hemisphere's surface, it remains unclear why certain symptoms—such as aphasia—are selectively affected in some patients while others are spared. A schematic of the common treatment paradigm and features of SDHs is provided in Figure 1.

**FIGURE 1.**
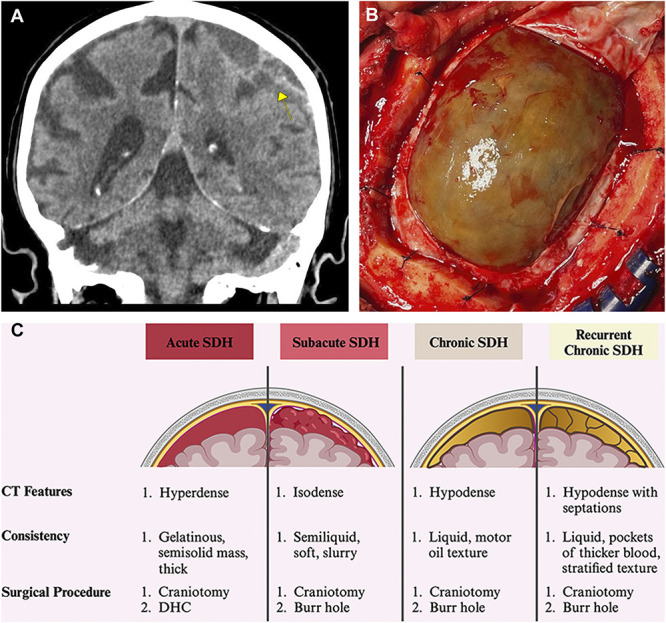
A schematic of common SDH features and surgical treatment. **A**, Coronal slice from a CT scan showing a chronic SDH (yellow arrow) with some subacute components. The hypodense, crescent-shaped lesion indicates old blood with septations, consistent with the chronic phase. **B**, Intraoperative photograph during a craniotomy for a chronic SDH, with the dura opened. A well-preserved subdural membrane is visible beneath the dura, and evacuation revealed a motor oil-like fluid, characteristic of cSDH. **C**, Schematic comparison of acute, subacute, chronic, and recurrent cSDH, summarizing their CT features, consistency (texture), and typical surgical procedures. Note the progression from hyperdense to hypodense appearance on CT and the shift in texture from gelatinous to liquefied across stages. cSDH, chronic SDH; CT, computed tomography; DHC, decompressive hemicraniectomy; SDH, subdural hematomas. *Figure 1C created in BioRender. Maeda, T. (2025)* https://BioRender.com/cn9wkhr.

Aphasia, in particular, can have a profound impact on an individual's quality of life. Patients may face challenges with physical and psychological functioning, interpersonal relationships, and loss of independence, leading to increased risk of emotional distress and social isolation.^11,12^ Yet, few studies have focused specifically on aphasia in the context of cSDH.^13-18^ It is still uncertain whether certain risk factors, such as older age, size, or anticoagulation therapy, increase the likelihood of developing aphasia. Moreover, the rate of recovery of aphasia after cSDH evacuation has not been previously reported, which would be valuable in counseling patients and setting expectations after surgery. Most existing studies on SDH outcomes rely on general scoring systems such as the Glasgow Outcome Scale (GOS) or modified Rankin Scale, but few have addressed the recovery of specific neurological deficits.^19-24^

In this study, we present the first detailed analysis of aphasia and its recovery in a predominantly elderly patient population who underwent cSDH evacuation. In addition, we explore the frequency of aphasia and other symptoms at the time of diagnosis, as well as the respective recoveries.

## METHODS

Patients consecutively treated at a tertiary academic center for SDH evacuation by a neurosurgeon were retrospectively identified from November 2013 to December 2021. The study included adult patients aged 18 and older. For patients with multiple admissions, only the initial encounter at the time of the primary surgery was recorded. Using International Classification of Diseases 9 and 10 billing codes, 689 patients were initially identified. After excluding 195 patients because of duplicate admissions, epidural hematoma evacuations, absence of surgery during admission, or acute SDH cases (157 patients), the focus narrowed to cSDH cases. SDHs were defined as chronic based on the presence of a primary hypodense, isodense, or mixed-density subdural collection.

**FIGURE 2.**
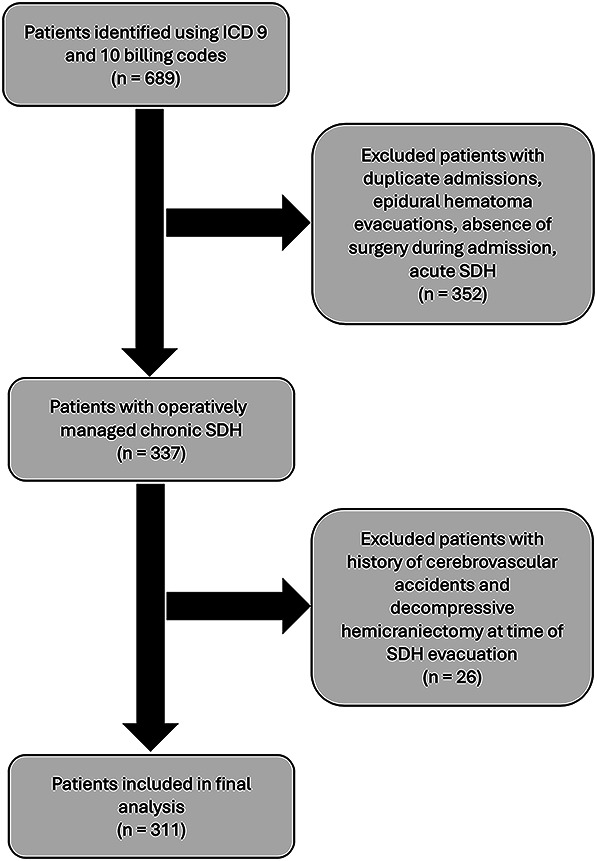
Flow-chart demonstrating study design. SDH, subdural hematomas.

Patients with a history of cerebrovascular accidents (23 patients) or those undergoing decompressive hemicraniectomy for cerebral edema at the time of SDH evacuation (3 patients) were also excluded to reduce confounding factors, leaving 311 patients for the final analysis (Figure 2).

Data were extracted from electronic medical records, including demographic information (age, sex), medical comorbidities, anticoagulant or antiplatelet use, surgical type (craniotomy vs burr hole), and cSDH characteristics (size, laterality, midline shift). Neurological examinations at admission and discharge were recorded, including the Glasgow Coma Scale (GCS) score. If the GCS score was not explicitly stated, it was calculated from the neurological examination findings by research staff. Aphasia (present or absent) was assessed based on bedside neurological examinations performed by neurosurgeons. Aphasia was classified as expressive (motor), receptive (sensory), or mixed (both expressive and receptive components), per neurosurgeon documentation. Only patients who had aphasia consistently before surgery, as shown in their examinations, were counted as having aphasia for the study. Patients with globally poor neurological examinations (eg, GCS 3-8) without focal deficits such as aphasia were not classified as aphasic; only 1 patient presented with a GCS score of 3 to 8. Aphasia status postevacuation was tracked during hospitalization and at discharge, alongside inpatient evaluation by a speech-language pathologist (SLP) evaluation. For 1 patient with aphasia who died inpatient, aphasia was recorded as present at death for data collection purposes.

Postoperative anti-seizure medication was administered as clinically indicated. Complete resolution of aphasia during admission was determined based on the presence or absence of aphasia listed in the physical examination at the time of discharge or the time of most recent follow-up for persistent cases, with improvement defined as reduced aphasia deficits compared with admission on physical examination. Patients with persistent symptoms were followed until their latest neurosurgical follow-up. GOS scores were calculated at discharge from neurological examination findings, per McMillan et al definitions provided, with scores of 1 to 3 classified as poor outcomes.^25^ Data collection and GOS scoring were conducted by research staff, including 2 senior medical students and a neurosurgery resident, all trained in GOS assessment. All patients in the study were assessed, with no exclusions due to inability to calculate a GOS score.

This retrospective clinical study received approval from the hospital's institutional review board, with a waiver of patient consent for data collection. Statistical analyses were performed using STATA 14 software (Stata-Corp LP). Patients were stratified by preoperative aphasia status (present or absent) and logistic regression was used to identify predictors of aphasia. Continuous variables were compared using Student's *t*-test for parametric data or the Wilcoxon rank-sum test for nonparametric data. Categorical variables were analyzed with the χ^2^ test, or Fischer's Exact test when expected frequencies in any category were <5. Univariate and multivariable logistic regression analyses were conducted with aphasia as the dependent variable. To avoid insufficient cases in a single group, right-sided and bilateral cSDHs were combined into 1 category and compared against left-sided cSDH. In addition, for this same reason, only hypertension was included from the medical comorbidities recorded. A significance level of *P* < .05 was applied throughout, and any missing data were left blank during the analysis.

## RESULTS

A total of 311 patients were included in this study. The mean age was 71 years, and most patients were men (76%, 236/311). The mean SDH size was 21 mm (range 7-40 mm), with midline shift present in 81% (249/311). A small craniotomy was the most commonly performed surgery, comprising 84% (261/311) of procedures, whereas the remainder were burr hole evacuations (16%, 50/311).

Ten percent of patients presented with aphasia as a symptom of cSDH (30/311). Most cases were holohemispheric (86.7%, 26/30) with the remainder localized to the frontal region. Expressive aphasia (25/30) predominated, whereas mixed aphasia occurred in the remaining patients (16.7%, 5/30). A comparison of demographics and SDH characteristics between patients with and without aphasia is presented in Table 1. Patients with aphasia had a significantly higher proportion of left-sided cSDH (73%, 22/30) than right (10%, 3/30) or bilateral (17%, 5/30). No significant differences were observed in age, sex, surgery type, medical comorbidities, antiplatelet use, or anticoagulation use. Outcomes were then compared between the cohort of patients with aphasia and those without. No significant differences were found including postoperative complications, length of stay, reoperation rate, discharge location, poor outcomes, mortality, and follow-up (Table 2).

**TABLE 1.** Patient Summary for Those Presenting With Aphasia Who Had a SDH Evacuated

Characteristics	Aphasia			
	Absent	Present	*P*-value	Total
No. of patients	281 (90)	30 (10)		311
Mean age, y (SD)	71 (15)	70 (15)	.83	71 (15)
Age range (y)	22-100	30-89		22-100
Males	214 (76)	22 (73)	.73	236 (76)
Mean SDH size, mm (SD)	21 (7)	20 (8)	.50	21 (7)
Mean symptom duration, d (SD)	9 (16)	11 (20)	.54	9 (16)
Laterality				
Right	110 (39)	3 (10)	**<.001**	113 (37)
Left	103 (37)	22 (73)		123 (40)
Bilateral	68 (24)	5 (17)		73 (23)
Midline shift present	228 (82)	21 (72)	.21	249 (81)
Surgery type				
Craniotomy	234 (83)	27 (90)	.44	261 (84)
Burr hole	47 (17)	3 (10)		50 (16)
Medical comorbidities				
Hypertension	108 (38)	15 (50)	.22	123 (40)
Diabetes mellitus	38 (14)	4 (13)	1	42 (14)
Coronary artery disease	35 (12)	3 (10)	1	38 (12)
Chronic heart failure	11 (4)	2 (7)	.47	13 (4)
Atrial fibrillation	24 (9)	2 (7)	.77	26 (8)
Aspirin use (Y)	85 (30)	6 (21)	.28	91 (29)
Plavix/brilinta (Y)	20 (7)	1 (3)	.70	21 (7)
Anticoagulation (Y)	45 (16)	4 (13)	1	49 (16)
GCS at time of arrival				
3-8	1 (0)	0		1 (0)
9-12	16 (6)	1 (3)	.81	17 (6)
13-15	264 (94)	29 (97)		293 (94)
GCS at time of discharge				
3-8	8 (3)	1 (3)		9 (3)
9-12	3 (1)	1 (3)	.57	4 (1)
13-15	270 (96)	28 (93)		298 (96)

GCS, Glasgow Coma Scale; SDH, subdural hematomas.

Characteristics between those with and those without aphasia were compared.

Bold values denote significant differences (*P*-value <.05).

**TABLE 2.** A Comparison of Patient Outcomes Between Those Who Presented With Aphasia Versus Those Who Did Not

Outcomes	Aphasia			
	Absent (n = 281)	Present (n = 30)	*P*-value	Total
Complications				
Urinary tract infection	7 (3)	0	1	7 (2)
Pneumonia	5 (2)	1 (3)	.46	6 (2)
Deep venous thrombosis/pulmonary embolism	1 (0)	0	1	8 (0)
Infection	11 (4)	1 (3)	1	12 (4)
Seizure	16 (6)	4 (13)	.12	20 (7)
Trach/PEG placed (Y)	13 (5)	1 (3)	1	14 (5)
Average length of stay days (SD)	9.5 (10)	15.5 (32)	.39	10.1 (14)
Reoperation	36 (13)	5 (17)	.57	41 (13)
Discharge location				
Nursing facility	52 (19)	3 (10)	.45	55 (18)
Rehab	77 (28)	12 (40)		89 (29)
Home	140 (51)	14 (47)		154 (50)
Hospice	8 (3)	1 (3)		9 (3)
Poor outcome at discharge (GOS 1-3)	22 (8)	1 (3)	.71	23 (7)
Inpatient mortality	9 (3)	1 (3)	1	10 (3)

DVT, deep vein thrombosis; GOS, Glasgow Outcome Scale; PE, pulmonary embolism; PEG, percutaneous endoscopic gastrostomy; PNA, pneumonia; trach, tracheostomy; UTI, urinary tract infection.

Poor outcomes were defined by a GOS 1 to 3 at the time of discharge.

Univariate logistic regression was then used to evaluate for risk factors of aphasia, including age, sex, SDH laterality (left vs right and bilateral), SDH size (mm), midline shift (mm), and hypertension (Table 3). Only left-sided SDH laterality was significantly associated with aphasia (odds ratio [OR] 4.89, 95% CI, 2.1-11.4, *P* < .001). A multivariable logistic model, adjusting for age, sex, and SDH laterality confirmed a similar association (OR 4.89, 95% CI, 2.09-11.41, *P* < .001).

**TABLE 3.** Logistic Regression Analysis for the Development of Aphasia in the Setting of a Chronic SDH

Logistic regression	Odds ratio	SE	*P*-value	95% CI
Age (y)	1	0.01	.93	0.98-1.03
Sex (male)	0.86	0.38	.73	0.37-2.02
cSDH laterality				
Right or bilateral	*Reference*	*Reference*	*Reference*	*Reference*
Left	4.83	2.07	**<.001**	2.07-11.23
SDH size (mm)	0.97	0.28	.33	0.92-1.03
Midline shift (mm)	0.95	0.04	.186	0.87-1.03
Hypertension	1.6	0.62	.22	0.75-3.41

cSDH, chronic subdural hematoma; SDH, subdural hematomas.

Bold values denote significant differences (*P*-value <.05).

**FIGURE 3.**
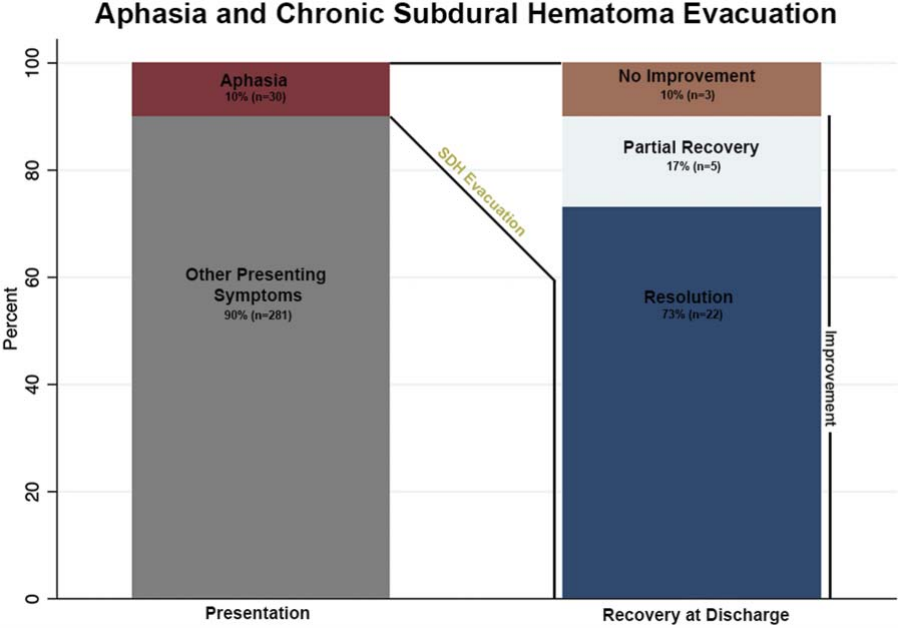
Distribution of patients who presented with aphasia and the rates of recovery after surgery at discharge.

After surgical evacuation, 90% (27/30) of patients had documented improvement in aphasia. Seventy-three percent (22/30) had complete resolution of aphasia by the time of discharge. Figure 3 summarizes the distribution of patients who presented with aphasia and the rates of recovery after surgery at discharge. Most of the patients (23/30) worked with SLP in the postoperative recovery period while inpatient. Of the 7 that did not, 1 died, 5 had resolved aphasia immediately after surgery, and 1 was transferred to an outside hospital.

Patients with unresolved aphasia at discharge (n = 8) had their neurological examination at the most recent neurosurgery follow-up visits recorded. Table 4 provides a summary of these patients. Of the 3 without any improvement from the time of surgery, the first had persistent expressive aphasia with dysarthric speech and a small amount of residual acute blood products on the postoperative scan; no MRI or further follow-up occurred after discharge. The second had expressive aphasia with dysarthric speech that persisted at 90 day follow-up. Postoperatively, an MRI revealed a small infarct along the left motor cortex attributed to a small vessel bleeding near the pia that was identified after irrigation and cauterized during surgery. The third patient had nonfluent aphasia characterized by paraphasic errors and word retrieval deficits that persisted at the 30 day follow-up. Computed tomography of the head after surgery showed expected postoperative changes; no MRI was completed after surgery.

**TABLE 4.** A Patient Summary of Those Without Resolution of Aphasia by the Time of Discharge

Patient no.	Age	Sex	SDH size (mm)	Laterality	Midline shift (mm)	Aphasia type	Postoperative improvement	Pertinent postoperative imaging findings	In Patient mortality	Resolution at FU	FU time (d)
1	86	M	16	L	7.7	Expressive	No	Residual blood products	No	No	0
2	84	M	24	L	0	Expressive	No	Infarct along L motor cortex	No	No	90
3	72	M	14	L	8	Expressive	No	No	No	No	30
4	89	M	11	L	8	Expressive	Yes	No	No	Yes	90
5	75	F	14	L	0	Expressive	Yes	Epidural hematoma (revision surgery)	No	Yes	180
6	30	M	18	L	0	Expressive	Yes	No	No	Yes	30
7	83	M	24	L	14	Expressive	Yes	Punctate Infarcts along L Anterior cerebral artery + Middle cerebral artery	Yes	No	0
8	77	M	L 12/R 16	BL	2	Expressive	Yes	No	No	No	90

BL, bilateral; F, female; FU, follow-up; L, left; M, male; SDH, subdural hematomas.

## DISCUSSION

This study contributes to the limited literature on surgical outcomes and recovery in patients presenting with aphasia after cSDH evacuation. With the projected increase in cSDHs incidence over the coming years, neurological deficits such as aphasia are likely to become more prevalent among neurosurgical patients, underscoring the need for targeted research and interventions.^26,27^ In our cohort, 10% of patients requiring cSDH evacuation exhibited preoperative aphasia, a condition known to impair quality of life by affecting emotional well-being and social functioning.^11,12^ Of these, 90% demonstrated improvement by discharge, including 73% who achieved complete resolution, a rate that rose to 83% at the most recent follow-up. No significant differences were observed in outcomes or complication rates—including length of stay, reoperation rate, discharge location, poor outcomes (GOS 1-3), or mortality—between patients with and without aphasia. These findings offer valuable prognostic insights for counseling patients and families about expected recovery trajectories after surgery.

Regarding risk factors, left-sided lesion laterality was the only significant predictor of aphasia (OR 4.89). This increased risk in patients with left-sided SDH aligns with language lateralization to the left cerebral hemisphere in most individuals.^28^ Injuries to key language areas—most commonly Broca's area, Wernicke's area, the supplementary motor area, or the arcuate fasciculus—in the dominant hemisphere often result in aphasia. However, damage to deep subcortical structures, such as the internal capsule, caudate nucleus, or thalamus, may also contribute.^29^

Assessing aphasia in patients with cSDH may be challenging at times, because altered mental status, confusion, or depressed consciousness may obscure or mimic aphasic symptoms. In this cohort, however, 94% (292/311) of patients presented with high GCS scores of 13 to 15, facilitating reliable aphasia evaluation. One patient with a GCS score of 3 to 8 was intubated at presentation and classified as without aphasia; no aphasia was noted postextubation during that admission. Among the 17 patients with GCS scores of 9 to 12, only 1 exhibited aphasia. Given their small proportion (5.5%, 18/311) of the study population, patients with lower GCS likely had minimal impact on the overall detection of aphasia. Nevertheless, subtle language impairments may exist in some patients or persist in others undetected during standard neurosurgical examinations, which are also subject to interobserver variability.^30^ For instance, Blaauw et al^31^ conducted cognitive testing with the Telephonic Interview of Cognitive Status-modified 3 months post-cSDH evacuation and found that 54% of patients exhibited impaired cognition, scoring significantly lower in verbal memory and language/reasoning domains. These findings suggest that people who did not present with aphasia (90%) may still have subtle language deficits, emphasizing the potential need for more sensitive, standardized assessments to accurately detect aphasia in this population.

Few studies have reported on aphasia in the context of cSDH (Table 5).^13-18^ Two of the largest investigations by Blaauw et al and Moster et al, examined aphasia among cSDH patients, including 45 and 9 patients, respectively.^13-18^ These studies primarily focused on transient neurological deficits (TNDs), such as transient aphasia, potentially linked to seizures or cortical spreading depolarization, which may differ mechanistically from continuous, nonepisodic aphasia observed in our cohort.^32,33^ For example, Moster et al^13^ described the TNDs that resolved within 30 minutes or less in the 3 listed case reports, although 1 did have “mild global aphasia” as well present on examination that resolved after surgery. Blaauw et al^18^ had 1 patient with a 24-hour duration TND, although the median was 10 minutes, suggesting variability in the duration and severity of language deficits. As such, the exact timeframe used to classify TNDs as transient vs persistent preoperative neurological deficits remains unclear in the literature. Consistent with our results, Blaauw et al^18^ found no difference in age, sex, recurrence rate, mortality, or functional outcome between those with TNDs-including aphasia-and those without.

**TABLE 5.** Summary of Previously Published Studies Investigating Chronic SDH and Aphasia

Authors	Location	Study type	Data date range	No. of patients presenting with aphasia	Recovery of aphasia (n (%))
Moster et al^13^	USA	SC case series	1976-1980	9	—
Dell et al^14^	USA	SC case series	1983	4	4 (100)
Kaminski et al^15^	USA	SC case series	1991	3	2 (66.7)
Rahimi et al^16^	USA	SC case report	2000	1	—
Kuwahara et al^17^	Japan	SC case report	2004	1	—
Blaauw et al^18^	Netherlands	MC retrospective analysis	2005-2019	45^a^	—

MC, multiple centers; SC, single series.

a Patients grouped as aphasia/dysphagia; recovery rates not recorded for this group.

In addition to TNDs, cognitive impairment is also prevalent and may intersect with aphasia. A systematic review of cognitive impairment recovery reported a preoperative prevalence of 61% that dropped to 18% postsurgery, reflecting a 71% resolution rate.^34^ By contrast, our study found a lower preoperative aphasia prevalence (10%), but a similar resolution rate (73% at discharge). This discrepancy in prevalence may suggest underdetection of subtle language deficits in our cohort, potentially misclassified as broader cognitive impairment, whereas the similar resolution rates highlight the overlap between aphasia and cognitive impairment in language-related deficits.

With some isolated exceptions, most of the patients with aphasia in this cohort were evaluated and treated by SLPs during their hospital admission. Speech and language therapy plays a valuable role in recovery. A Cochrane review by Brady et al^35^ found that patients randomized to formal SLP intervention, compared with standard care, showed significant improvements in functional communication, reading, writing, and expressive language. Although this evidence stems from a poststroke population, many of these strategies are likely applicable to patients with cSDH. For example, Constraint-Induced Language Therapy, an intensive approach, restricts nonverbal communication alternatives to compel the brain to re-engage damaged language networks, promoting neuroplasticity.^36^

An intriguing finding in our study was the predominance of expressive aphasia, with no patients exhibiting isolated receptive aphasia. This may reflect selective vulnerability of motor neurons to injury in the content of cSDH. Motor neurons, which are relatively large and have high metabolic demands, may be more susceptible to the toxic effects of extravasated blood.^37^ Whole blood contains thrombin, hemoglobin, and free iron at concentrations far exceeding those required to induce neuronal death, with levels in whole blood reaching 7.5 times the thrombin, ∼1000 times the hemoglobin, and 1 to 3 times the free iron concentrations needed for half-maximal neuronal death.^38^ In addition, the large axons of motor neurons require a significant amount of energy, consuming 1 adenosine triphosphate molecule for every 8-nm displacement of cargo during axonal transport.^39^ This high dependence for continuous energy makes them especially vulnerable to energetic stress such as that may be induced by the mass effect and ischemia associated with cSDH.^40^ The predominance of expressive aphasia, which involves motor control of speech articulation, may also be influenced by the anatomic distribution of the hematomas in our cohort, which were more commonly located over the frontal lobe, potentially affecting the primary motor cortex and Broca's area. Alternatively, the absence of isolated receptive aphasia may suggest that sensory neurons in Wernicke's area, typically located in the temporal lobe, are less affected by the hematoma's location or have lower metabolic demands, although this requires further investigation.

Limitations of this study include its retrospective design, which may introduce selection bias, and its dependence on the accuracy of clinical documentation. Although most patients improved after cSDH evacuation, outpatient speech-language pathology interventions may have further enhanced aphasia recovery. However, the absence of documentation from contracted speech-language pathology facilities in the medical charts, coupled with loss to follow-up, precluded assessment of this contribution. Furthermore, the small number of aphasia cases (n = 30) likely underpowered the study, limiting detection of weaker associations in the logistic regression analysis. Similarly, with only 8 patients (27%) having residual aphasia at discharge, we were unable to robustly compare factors influencing recovery, such as age, hematoma characteristics, or SLP intervention, between those who did and did not regain full language function. Last, it would have been informative if all patients with persistent aphasia postoperatively had undergone MRI to evaluate for the presence of an infarct, which could explain incomplete recovery; however, this was not routinely performed. Future studies with prospective designs, standardized documentation, and larger sample sizes are needed to address these limitations and better elucidate the factors influencing aphasia recovery in cSDH patients.

## CONCLUSION

Despite being a relative common pathology with a growing incidence, very few studies have specifically reported on aphasia associated with cSDHs. In this analysis, aphasia was found present in 10% of the 311 patients who underwent cSDH evacuation. Among these patients, all exhibited an expressive (83%) or mixed phenotype (17%), but none presented exclusively with receptive aphasia. With the exception of left-sided laterality, no patient characteristics, medical comorbidities, or antithrombotic medication use was identified as a risk factor of aphasia. After surgery, the majority improved and nearly three-quarters resolved by discharge, which increased to 83% at the most recent follow-up. These findings represent the first and largest series to address this topic, which may help guide treatment decisions and counseling for patients.

## COMMENTS

Almost 35% of all patients admitted to the Shock Trauma Center with blunt traumatic brain injury carry an acute subdural hematoma. Many of these patients are followed conservatively expecting natural liquefaction and resorption of their subdural blood. Complete resorption of acute SDH may not happen. Traumatic Injury to the dural border cells allows CSF mixed with blood and its toxic components including thrombin, iron and hemoglobin to enter the subdural space starting an acute inflammatory cascade activating cytokines and interleukins followed by activation of angiogenetic factors such as VEGF, COX-2, TGF-β1 and PDGF. Neovascularization predisposes repeated episodes of hemorrhage in the subdural space. Pathogenetically this process results in a highly vascular parietal membrane and an avascular plastic-like visceral membrane and multiseptated cavities filled with organized blood (Figure 1). Of significance, the visceral membrane does not have much adhesion to the subjacent cortex and in fact when the arachnoid adhesions are separated, the entire parietal and visceral membranes could be removed completely (Figure 1).

The present submission suggests the compressive tendency of chronic SDH rather than irreversible cortical damage. As such, reversal of motor dysphasia in nearly 85% of the patients is not unexpected. One could argue that even middle meningeal artery embolization and gradual regression of the size of the subdural hematoma can herald progressive improvement of motor dysphasia. Finally, the disability produced by transient dysphasia might be considered a process separate from the Frailty Index which is high in this elderly group of patients.

**Bizhan Aarabi**

*Baltimore, Maryland, USA*

## References

[1] Nouri A Gondar R Schaller K Meling T. Chronic subdural hematoma (cSDH): a review of the current state of the art. Brain Spine. 2021;1:100300. 36247395 10.1016/j.bas.2021.100300 PMC9560707

[2] Neifert SN Chaman EK Hardigan T Increases in subdural hematoma with an aging population-the future of American cerebrovascular disease. World Neurosurg. 2020;141:e166-e174. 32416236 10.1016/j.wneu.2020.05.060

[3] Dossett LA Riesel JN Griffin MR Cotton BA. Prevalence and implications of preinjury warfarin use: an analysis of the national trauma databank. Arch Surg. 2011;146(5):565-570. 21242422 10.1001/archsurg.2010.313

[4] Chen H Colasurdo M Malhotra A Gandhi D Bodanapally UK. Advances in chronic subdural hematoma and membrane imaging. Front Neurol. 2024;15:1366238. 38725642 10.3389/fneur.2024.1366238 PMC11079242

[5] Taylor CA Bell JM Breiding MJ Xu L. Traumatic brain injury-related emergency department visits, hospitalizations, and deaths - United States, 2007 and 2013. MMWR Surveill Summ. 2017;66(9):1-16. 10.15585/mmwr.ss6609a1 PMC5829835 28301451

[6] Vespa J Medina L Armstrong DM. Demographic Turning Points for the United States: Population Projections for 2020 to 2060. U.S. Census Bureau; 2020.

[7] UN Department of Economic and Social Affairs. Leaving No One Behind in an Ageing World. New York: United Nations Publications; 2023.

[8] Tenny S Thorell W. Intracranial Hemorrhage. StatPearls [Internet]; 2024. 29262016

[9] Blaauw J Meelis GA Jacobs B Presenting symptoms and functional outcome of chronic subdural hematoma patients. Acta Neurol Scand. 2022;145(1):38-46. 34448196 10.1111/ane.13518

[10] Nisson PL Francis J Michel M Focal motor weakness and recovery following chronic subdural hematoma evacuation. J Neurosurg. 2024;141(6):1739-1746. 38875718 10.3171/2024.4.JNS24121

[11] Spaccavento S Craca A Del Prete M Quality of life measurement and outcome in aphasia. Neuropsychiatr Dis Treat. 2014;10:27-37. 24368886 10.2147/NDT.S52357 PMC3869916

[12] Filipska-Blejder K Zielińska J Zieliński M Wiśniewski A Ślusarz R. How does aphasia affect quality of life? Preliminary reports. J Clin Med. 2023;12(24):7687. 38137755 10.3390/jcm12247687 PMC10744265

[13] Moster ML Johnston DE Reinmuth OM. Chronic subdural hematoma with transient neurological deficits: a review of 15 cases. Ann Neurol. 1983;14(5):539-542. 6651241 10.1002/ana.410140508

[14] Dell SO Batson R Kasdon DL Peterson T. Aphasia in subdural hematoma. Arch Neurol. 1983;40(3):177-179. 6830461 10.1001/archneur.1983.04050030071015

[15] Kaminski HJ Hlavin ML Likavec MJ Schmidley JW. Transient neurologic deficit caused by chronic subdural hematoma. Am J Med. 1992;92(6):698-700. 1605153 10.1016/0002-9343(92)90790-i

[16] Rahimi AR Poorkay M. Subdural hematomas and isolated transient aphasia. J Am Med Dir Assoc. 2000;1(3):129-131. 12818026

[17] Kuwahara S Miyake H Koan Y [A case of organized chronic subdural hematoma presented with transient neurologic deficits]. No To Shinkei. 2004;56(4):355-359. 15237729

[18] Blaauw J den Hertog HM van Zundert JM Transient neurological deficit in patients with chronic subdural hematoma: a retrospective cohort analysis. J Neurol. 2022;269(6):3180-3188. 34999957 10.1007/s00415-021-10925-8

[19] Amirjamshidi A Eftekhar B Abouzari M Rashidi A. The relationship between Glasgow coma/outcome scores and abnormal CT scan findings in chronic subdural hematoma. Clin Neurol Neurosurg. 2007;109(2):152-157. 16949734 10.1016/j.clineuro.2006.07.007

[20] Ran KR Alfonzo Horowitz M Liu J Evaluation of the Glasgow Coma Scale-Pupils score for predicting inpatient mortality among patients with traumatic subdural hematoma at United States trauma centers. J Neurosurg. 2024;141(4):908-916. 38701532 10.3171/2024.2.JNS232695

[21] Amirjamshidi A Abouzari M Eftekhar B Outcomes and recurrence rates in chronic subdural haematoma. Br J Neurosurg. 2007;21(3):272-275. 17612917 10.1080/02688690701272232

[22] Amirjamshidi A Abouzari M Rashidi A. Glasgow Coma Scale on admission is correlated with postoperative Glasgow Outcome Scale in chronic subdural hematoma. J Clin Neurosci. 2007;14(12):1240-1241. 17382549 10.1016/j.jocn.2006.03.030

[23] Leitgeb J Mauritz W Brazinova A Outcome after severe brain trauma due to acute subdural hematoma. J Neurosurg. 2012;117(2):324-333. 22631691 10.3171/2012.4.JNS111448

[24] Weimer JM Gordon E Frontera JA. Predictors of functional outcome after subdural hematoma: a prospective study. Neurocrit Care. 2017;26(1):70-79. 27230968 10.1007/s12028-016-0279-1

[25] McMillan T Wilson L Ponsford J Levin H Teasdale G Bond M. The Glasgow Outcome Scale - 40 years of application and refinement. Nat Rev Neurol. 2016;12(8):477-485. 27418377 10.1038/nrneurol.2016.89

[26] Adhiyaman V Chattopadhyay I Irshad F Curran D Abraham S. Increasing incidence of chronic subdural haematoma in the elderly. QJM. 2017;110(6):375-378. 28069915 10.1093/qjmed/hcw231

[27] Rauhala M Helén P Huhtala H Chronic subdural hematoma-incidence, complications, and financial impact. Acta Neurochir (Wien). 2020;162(9):2033-2043. 32524244 10.1007/s00701-020-04398-3 PMC7415035

[28] Riès SK Dronkers NF Knight RT. Choosing words: left hemisphere, right hemisphere, or both? Perspective on the lateralization of word retrieval. Ann N Y Acad Sci. 2016;1369(1):111-131. 26766393 10.1111/nyas.12993 PMC4874870

[29] Kuljic-Obradovic DC. Subcortical aphasia: three different language disorder syndromes? Eur J Neurol. 2003;10(4):445-448. 12823499 10.1046/j.1468-1331.2003.00604.x

[30] Hansen M Sindrup SH Christensen PB Olsen NK Kristensen O Friis ML. Interobserver variation in the evaluation of neurological signs: observer dependent factors. Acta Neurol Scand. 1994;90(3):145-149. 7847053 10.1111/j.1600-0404.1994.tb02697.x

[31] Blaauw J Hertog HMD Holl DC The cognitive status of chronic subdural hematoma patients after treatment: an exploratory study. Acta Neurochir (Wien). 2023;165(3):701-709. 36752891 10.1007/s00701-023-05508-7 PMC10006248

[32] Blaauw J Zundert JMV den Hertog HM Pathophysiology of transient neurological deficit in patients with chronic subdural hematoma: a systematic review. Acta Neurol Scand. 2022;145(6):649-657. 35355247 10.1111/ane.13617

[33] Levesque M Deacon C Adam S Iorio-Morin C. Cortical spreading depolarization in chronic subdural hematoma: bridging the gap. Can J Neurol Sci. 2021;48(1):31-37. 32631474 10.1017/cjn.2020.128

[34] Blaauw J Boxum AG Jacobs B Prevalence of cognitive complaints and impairment in patients with chronic subdural hematoma and recovery after treatment: a systematic review. J Neurotrauma. 2021;38(2):159-168. 32873143 10.1089/neu.2020.7206

[35] Brady MC Kelly H Godwin J Enderby P Campbell P. Speech and language therapy for aphasia following stroke. Cochrane Database Syst Rev. 2016;2016(6):Cd000425. 27245310 10.1002/14651858.CD000425.pub4 PMC8078645

[36] Raymer AM Roitsch J. Effectiveness of constraint-induced language therapy for aphasia: evidence from systematic reviews and meta-analyses. Am J Speech Lang Pathol. 2023;32(5s):2393-2401. 36668725 10.1044/2022_AJSLP-22-00248

[37] Vandoorne T De Bock K Van Den Bosch L. Energy metabolism in ALS: an underappreciated opportunity? Acta Neuropathol. 2018;135(4):489-509. 29549424 10.1007/s00401-018-1835-x PMC5978930

[38] Stokum JA Cannarsa GJ Wessell AP Shea P Wenger N Simard JM. When the blood hits your brain: the neurotoxicity of extravasated blood. Int J Mol Sci. 2021;22(10):5132. 34066240 10.3390/ijms22105132 PMC8151992

[39] Hua W Young EC Fleming ML Gelles J. Coupling of kinesin steps to ATP hydrolysis. Nature. 1997;388(6640):390-393. 9237758 10.1038/41118

[40] Le Masson G Przedborski S Abbott LF. A computational model of motor neuron degeneration. Neuron. 2014;83(4):975-988. 25088365 10.1016/j.neuron.2014.07.001 PMC4167823

